# Impact of Personality Traits on Coping Styles and Mental Health of Pakistani Medical Students

**DOI:** 10.1192/j.eurpsy.2026.10766

**Published:** 2026-07-31

**Authors:** A. Manzoor, S. Ijaz, S. M. Omer, S. Q. Bokhari, A. Butt, Q. M. Bokhari, A. Waheed, A. Anjum, S. Akhtar

**Affiliations:** 1 Psychiatry & Behavioural Sciences, Services Hospital Lahore; 2 Clinical Psychology, University of Central Punjab; 3 Psychiatry, Fatima Jinnah Medical University; 4 Psychiatry, Services Institute of Medical Sciences, Lahore; 5 Psychiatry & Behavioural Sciences, Rai Foundation Medical College, Sargodha; 6 Psychiatry, Punjab Institute of Mental Health, Lahore, Pakistan

## Abstract

**Introduction:**

Medical education is a highly demanding and stressful program, often leading to elevated stress, depression, and anxiety among students. Personality traits influence coping strategies, which in turn affect mental health outcomes. Understanding these relationships can help in designing the interventions to promote psychological well-being.

**Objectives:**

This study examines the impact of personality traits on coping styles and mental health among Pakistani medical students, identifies the coping strategies most frequently used, and assesses the relationship between adaptive and maladaptive coping strategies and psychological well-being. It also aims to help students understand how personality profiles impact stress management, facilitating the development of targeted support programs.

**Methods:**

Using a correlational cross-sectional design, data were collected from MBBS students at the Public Sector Medical College in Lahore, Pakistan, through purposive sampling. Participants completed the Big Five Personality Test, the Brief COPE Inventory, and the General Health Questionnaire-12. Data were analyzed using SPSS with Pearson correlation and regression analysis.

**Results:**

A Pearson correlation revealed a significant positive association between Neuroticism and avoidant coping, r(77) = .28, p = .015, indicating that students higher in Neuroticism tended to engage more in avoidant coping. Extraversion was positively associated with problem-focused coping, r(77) = .35, p = .002, suggesting that students higher in Extraversion were more likely to use problem-focused strategies. Openness to Experience was also positively correlated with problem-focused coping, r(77) = .29, p = .009, showing that students with higher openness scores engaged more in problem-focused coping. A multiple regression examined which Big Five traits predicted better general mental health. Results indicated that only Agreeableness was a significant predictor, β = .39, t(71) = 3.31, p = .001, such that higher Agreeableness was associated with better general health.

**Conclusions:**

Findings highlight the significant role personality traits play in shaping coping and mental health among Pakistani medical students. These results underscore the need for interventions in medical education that address stress management training while considering personality profiles. Effective coping strategies are crucial for managing challenges, achieving academic success, and maintaining emotional well-being. By fostering adaptive coping tailored to individual traits, medical institutions can better support the well-being of future healthcare professionals.

**Disclosure of Interest:**

None Declared

